# Improved Treatment Outcomes With Modified Induction Therapy in Acute Myeloid Leukemia (AML): A Retrospective Observational Study From a Regional Cancer Center

**DOI:** 10.7759/cureus.53303

**Published:** 2024-01-31

**Authors:** Asif Iqbal, Manas Dubey, Amritjot Singh Randhawa, Duncan Khanikar, Munlima Hazarika, Partha S Roy, Chayanika Dutta, Suhani Barbhuiyan, Roopam Deka

**Affiliations:** 1 Adult Hematology, Dr. Bhubaneswar Borooah Cancer Institute, Guwahati, IND; 2 Medical Oncology, Dr. Bhubaneswar Borooah Cancer Institute, Guwahati, IND; 3 Pediatric Oncology, Dr. Bhubaneswar Borooah Cancer Institute, Guwahati, IND; 4 Oncopathology, Haematopathology, Dr. Bhubaneswar Borooah Cancer Institute, Guwahati, IND

**Keywords:** induction outcomes, induction-related mortality, hypomethylating agents (hmas), relapse/remission rate, induction therapy, acute myeloid leukemia (aml)

## Abstract

Background: The aggressive, genetically diverse group of malignant illnesses known as acute myeloid leukemia (AML) is characterized by clonally related myeloblast invasion of the bone marrow, blood, and other organs. The treatment regimen plays a crucial role in the management of AML, and it is associated with poor overall survival and enhanced risk of relapse. Induction therapy with a 7+3 DA regimen (daunorubicin + ara-C) has been the treatment of choice for young and fit patients.

Objective: To evaluate the effect of dose modification in young and fit patients for a modified treatment regimen.

Methods: This was a retrospective, observational study of AML patients to analyze the outcomes of modified induction therapy in AML patients enrolled at Dr. B. Borooah Cancer Institute, Guwahati, Assam, India, from October 2021 to March 2022. The outcomes of modified induction therapy with intensive chemotherapy (modified 7+3 DA) and low-intensity chemotherapy decitabine (10 days) and venetoclax + azacytidine (seven days) were considered after the first two cycles or 60 days, whichever was earlier.

Results: Data from 31 patients with de-novo AML was analyzed; the median age of the patients was 41 years (range: 2-71 years), and the male-to-female ratio was 1.8. There were seven patients in the pediatric age group (2-13 years), and 19%, 65%, and 13% of patients belonged to favorable, intermediate, and high-risk groups, respectively. With regards to modified induction therapy (n=31), 20 (65%) patients received modified “7+3 DA”, nine (29%) received hypomethylating agents (HMA, decitabine only), and two patients received HMA (azacitidnie) + venetoclax. Additionally, 23/31 patients completed at least two cycles of induction therapy. Overall, 60 day-induction mortality was 13%, and the complete remission (CR) and partial remission (PR) rates were 48% and 26%, respectively. In patients who received modified “7+3 DA”, the CR rate was 55%.

Conclusions: The notable reduction in deaths due to infections observed in our study suggests that centers with limited resources for preventing neutropenic complications during induction therapies in AML patients could consider adopting this modified regimen.

## Introduction

Leukemia is a group of hematological malignancies in which there is an uncontrolled proliferation of leukemic cells replacing normal hematopoietic elements by abnormal cells in bone marrow. Acute myeloid leukemia (AML) in India has a poor prognosis and treatment and constitutes 1-2% of all cancer types [1]. It accounts for a major proportion of hematopoietic malignancies worldwide. Seventy percent of adult leukemias are AML. As per GLOBOCAN 2020, the worldwide estimates of cancer incidence and mortality for developing countries in 2020 revealed 269,503 and 205,016 new cases of leukemia in males and females, respectively. Of these, the estimated deaths were 176,000 and 132,000 in males and females, respectively [2]. Therefore, timely diagnosis and initiation of induction chemotherapy are required for complete remission (CR). In India, AML constitutes 1-2% of all cancer incidences [3]. However, with an overall five-year survival (OS) of 28.3%, the prognosis of AML remains poor. The OS is 40-50% in patients younger than 50 years of age with newly diagnosed AML, whereas for the elderly, it is only 5-10% [4-6].

Historically, the standard treatment strategy for AML has included a decision between intensive or less-intensive induction, followed either by post-remission stem cell transplantation or consolidation chemotherapy. Treatment decisions are based on the patient’s performance status, associated comorbidities, and age, as well as biological features of the leukemia that are predictive for treatment-related outcomes [7]. The intensive treatment phase of AML consists of two parts: induction of remission and consolidation therapy. Intensive chemotherapy is deemed for young and fit patients, while less intensive therapy with hypomethylating agents (HMAs) with or without newer agents is generally preferred for elderly or unfit patients.

The most commonly used intensive induction therapy for untreated AML, which involves the combination of anthracycline (mainly daunorubicin) plus cytarabine, also well-known as the "7+3" regimen, which comprises intravenous daunorubicin administered at a dose of 45-60 mg/m^2^ for three days and cytarabine at a dose of 100-200 mg/m^2^ administered by continuous IV infusion for seven days, has demonstrated CR rates ranging from 50% to 75% [8,9]. However, attempts to improve outcomes through the addition of other drugs to daunorubicin and cytarabine [10] or intensification of cytarabine doses have not shown significant benefits [11-13]. After achieving remission, high-dose cytarabine is commonly employed as consolidation therapy. Additionally, intermediate-dose cytarabine alone or in combination with other drugs is widely used for post-remission therapy.

In developed countries, the induction mortality is less than 5% [14]. However, in developing nations such as India, there are enormous challenges for treatment because of limited resources, high leukemic burden, baseline infections, poor general conditions, and poor socio-economic status, leading to a high mortality rate of 25% in patients receiving induction therapy; 88% of these deaths were infection-related [15]. Factors responsible for a higher mortality rate in AML in India were the presence of infections at presentation, infections with multi-drug resistant organisms (MDRO), invasive fungal infections at baseline, and the significant time interval between diagnosis and the start of the induction phase of chemotherapy [16].

Given the gravity of this situation and the urgent need to improve induction therapy outcomes for AML patients in India, this study aims to assess the effect of dose modification in young and fit patients undergoing modified treatment regimens. By evaluating the outcomes of such modifications in a real-world setting at our regional cancer center (RCC), we hope to identify potential solutions and strategies to optimize induction therapy, enhance treatment success, and reduce induction mortality rates in the context of resource-limited settings. This study aims to address the limitations and challenges associated with the current treatment strategies, especially in resource-limited settings such as India. By investigating the outcomes of modified induction regimens at our RCC, we seek to optimize treatment approaches and potentially improve remission rates, thus contributing to better overall survival for AML patients.

## Materials and methods

This is a retrospective, observational study of patients with de novo AML who were treated during the study period of six months at Dr. Bhubaneshwar Borooah Cancer Institute (BBCI), Guwahati, Assam, India (Oct 2021-March 2022). Patients with confirmed de novo AML (bone marrow morphology and flow cytometry (FCM)) were included. The study population included patients within the age group of one to 80 years who had a confirmed diagnosis of de novo AML through bone marrow morphology and FCM at BBCI. Patients were excluded from the study if they met any of the following criteria: (1) diagnoses of AML not confirmed by FCM at BBCI, resulting in uncertainty about the disease type; (2) patients with mixed phenotypic acute leukemia; (3) therapy-related AML cases, as these patients might have undergone previous treatment that could influence their response to induction therapy; (4) patients diagnosed with acute promyelocytic leukemia; (5) individuals who passed away before the initiation of induction chemotherapy; and (6) patients who left the hospital before the completion of induction chemotherapy.

Patients were categorized based on their age and physical condition. Those who were considered young and fit had an age of 60 years or younger and a performance status (PS) of 2 or lower. On the other hand, elderly and unfit patients were defined as those above the age of 60 or with a PS higher than 2. Additionally, the patients were stratified as high risk according to the ELN 2017 Risk stratification criteria. The high-risk category is important for assessing the outcomes and understanding the impact of the different induction therapies on this specific group of patients.

The treatment regimens for patients were based on their specific characteristics and risk stratification as follows (Table 1): (1) young/fit - standard/intermediate risk (SR/IR) patients: This group received a modified induction therapy regimen that is the "7+3 DA" protocol, which involved daunorubicin (45 mg/m^2^, days one to three) and cytarabine (200 mg/m^2^ on days one to two) as a continuous infusion, followed by 100 mg/m^2^ 12 hourly from days three to seven for a total of two induction cycles; (2) elderly/unfit patients: patients in this group were treated with decitabine (20 mg/m^2^, days one to 10) for a total of two induction cycles at an interval of 28 days; and (3) high-risk (HR) patients: high-risk patients received a combination of azacytidine (75 mg/m^2^) and venetoclax (100 mg/m^2^) from days one to seven, along with posaconazole for two induction cycles.

**Table 1:** The treatment protocol administered in young/fit patients and elderly/ unfit and high risk patients.

Protocol	Modification	No. of cycles
“7+3” (Young/Fit –SR/IR patients)	Daunorubicin @45mg/m^2^ (days 1 to 3) with ARA-C @100 mg/m^2^ CIVI for Day 1 to 2 f/b 100 mg/m^2^ over 2 hours 12 hourly from day 3 to 7	Two
Inj Decitabine (Elderly/Unfit patients)	@ 20 mg/m^2^ day 1 to 10 repeating after 28 days	Two
Inj Azacytidine + Venetoclax (HR patients)	Aza- @75 mg/m^2^ (day1 to 7) + Venetoclax100 mg (day 1 to 14 with Posaconazole) repeating after count recovery or 35 days (whichever is earlier)	Two

Study endpoints and assessments (study outcomes)

This research aimed to collect data on the proportion of patients achieving CR, the proportion achieving partial response (PR), and the induction mortality rate. Two primary endpoints to evaluate the outcomes of induction therapy were CR and PR. Additionally, the cause of induction-related deaths was considered as a secondary endpoint was also assessed. CR and PR are characterized by bone marrow aspiration with blasts <5% and >5%, respectively, after completing two cycles of induction therapy but transfusion independence. Induction-related deaths (induction mortality) are defined as deaths occurring due to any cause within 60 days of starting the first induction cycle. Induction therapy was administered to achieve these outcomes, and it included three treatment options: two cycles of the modified "7+3" regimen, or first two cycles of decitabine, or first two cycles of venetoclax + azacytidine. The secondary endpoint of the study aimed to identify the causes of induction-related deaths, which were likely monitored and recorded during the research. The outcomes of modified induction therapy with intensive chemotherapy were assessed after the completion of the first two induction cycles or within 60 days, whichever came earlier. In cases where low-intensity chemotherapy (venetoclax + azacytidine) or decitabine alone was administered, outcomes were analyzed after the completion of the first two cycles.

Statistical analysis

The statistical analysis involved summarizing data from all clinical assessments, as well as demographic and baseline characteristics, using descriptive statistics. For categorical variables, frequencies and percentages (%) with two-sided 95% confidence intervals (CI) were presented. Continuous variables were summarized with counts, means, standard deviations, medians, minimum values, and maximum values. The data analysis was performed using Statistical Product and Service Solutions (SPSS, version 27.0; IBM Corp., Armonk, NY).

## Results

Patient disposition and characteristics

**Figure 1:**
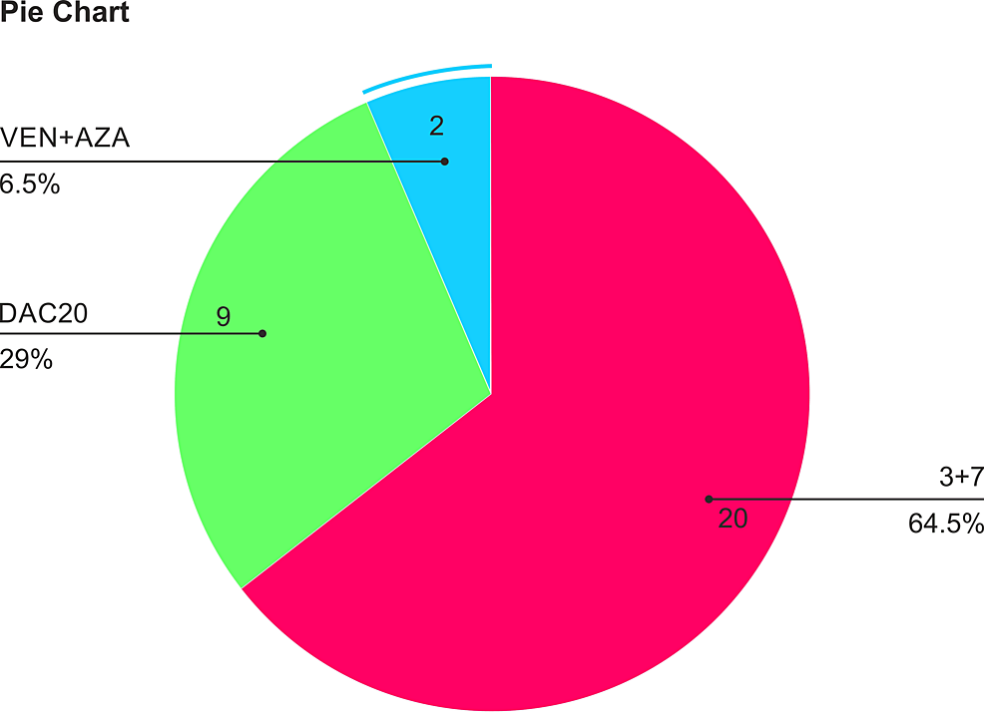
Risk stratification: 31 patients included in the study and divided into the following groups - favorable (19%), intermediate (65%), and high risk (13%).

Between October 2021 and March 2022, a total of 31 de-novo AML patients were identified in this study. The median age of patients was 41 years (range: 2-71 years), and the male-to-female ratio was 1.8. Seven patients belonged to the pediatric age group (2-13yrs). Out of 31 patients, 19%, 65%, and 13% were in the favorable, intermediate, and high-risk groups, respectively (Figure 1).

**Figure 2:**
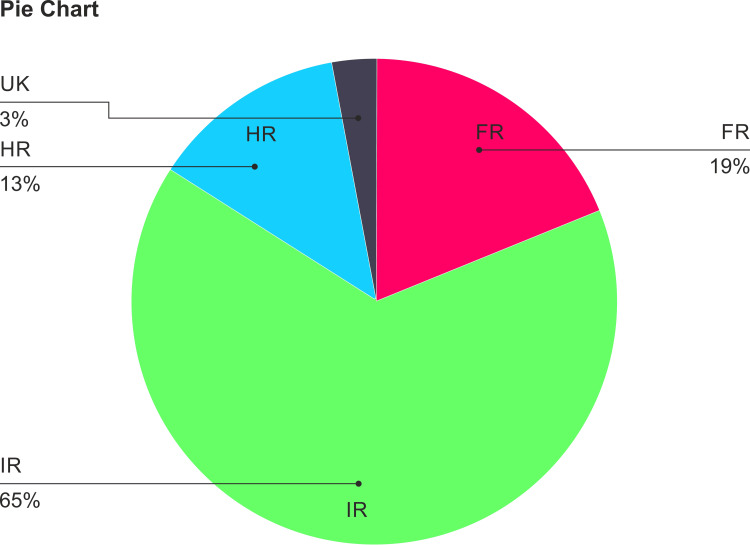
Treatment allocation: out of 31 patients, 20 (65%) patients received modified “7+3,” nine (29%) received HMA (decitabine), and two patients received HMA and venetoclax.

With regards to modified induction therapy (n=31), 20 (65%) patients received modified “7+3,” nine (29%) received HMA (decitabine) only, and two patients received HMA+ venetoclax (Figure 2). Out of 31 patients, 23 completed at least two cycles of induction therapy.

Study outcomes and mortality rates in AML patients undergoing modified induction therapies

Four patients died during induction therapy. All deaths were observed in patients who were treated with the modified “7+3” protocol. All deaths were due to infectious causes. Multidrug-resistant Gram-negative bacilli (MDR-GNB) were the most common cause of death in our setting. The overall 60-day induction mortality was 13%, and CR and PR rates were 48% and 26%, respectively. In patients who received modified “7+3,” the CR rate was 55%. No induction-related deaths were observed in the pediatric population. The group-wise mortality rates and remission rates have been summarized in Table 2.

**Table 2:** The mortality and remission rates in the respective groups.

Group (n=31)	Completed 2 Cycles of Induction Therapy	Induction Deaths, n (%)	CR, n (%)	PR, n (%)
Modified “7+3” (n=20)	14 (70%)	4 (20%)	11 (55%)	5 (25%)
Decitabine (n=9)	7 (77%)	Nil	3 (33%)	6 (66.7%)
Ven +Aza (n=2)	2 (100%)	Nil	2 (100%)	-

## Discussion

The management of AML remains a challenging endeavor, particularly in resource-limited settings in developing countries such as India. In this retrospective observational study, we aimed to evaluate the outcomes of modified induction therapies in AML patients treated at our regional cancer center between October 2021 and March 2022. The management of AML relies heavily on the treatment regimen, which is known to be associated with poor overall survival and a heightened risk of relapse. Induction therapy using the 7+3 regimen has shown favorable outcomes in young and fit patients. However, during the induction phase of AML treatment in India, a major challenge is the increased incidence of deaths caused by MDR organisms and fungal infections due to induction therapy.

Therefore, in the present study, we aimed to address these challenges associated with high induction mortality rates in AML patients by implementing modified induction therapies. Additionally, this approach was motivated by the findings of phase 3 randomized trials and recent studies that demonstrated improved outcomes with intensified induction regimens [17-19]. Moreover, the use of intensive induction chemotherapy in elderly patients (>65 years) with AML is often limited due to their increased susceptibility to adverse genomic features and resistance to treatments. Consequently, alternative treatment options are required for this population, and HMAs such as decitabine and azacytidine have emerged as viable choices. These HMAs have emerged as a promising therapeutic option for this vulnerable population, providing an alternative to intensive induction chemotherapy and potentially improving treatment outcomes [20]. The use of HMAs in combination with venetoclax as induction therapy for high-risk patients with adverse cytogenetics is justified by compelling evidence from a study conducted by DiNardo et al. [21]. In this study, 431 patients were enrolled, and those receiving azacitidine-venetoclax demonstrated a significant improvement in OS with a median OS of 14.7 months, compared to 9.6 months in the control group (azacitidine-placebo). The hazard ratio for death was 0.66, indicating a favorable survival benefit with the combination therapy [21]. In another study, 67% of patients achieved CR or CR with incomplete count recovery (CRi), with venetoclax of 400 mg + HMA cohort, exhibiting a CR + CRi rate of 73% [22]. These findings suggest that HMAs with venetoclax hold promise as an effective induction strategy for high-risk AML patients, particularly those with poor-risk cytogenetics and advanced age, warranting consideration in clinical practice.

In the present study, we included 31 de-novo AML patients, spanning a broad age range from two to 71 years. This diversity in age groups allowed for a comprehensive analysis of the modified induction therapy outcomes across different patient categories. Notably, the majority of patients fell under the intermediate risk category, suggesting a substantial representation of patients with moderate prognostic factors. The distribution of modified induction therapies revealed that the "7+3" regimen was the most frequently administered regimen, primarily targeting young and fit patients. On the other hand, HMAs, particularly decitabine, were utilized as an alternative for elderly or unfit individuals.

Induction therapy is recognized as the most intensive phase of AML treatment, and its success is vital for achieving remission [23]. However, our study revealed that induction-related mortality was a concern, with a mortality rate of 13% within 60 days of starting induction therapy. All induction-related deaths occurred in patients treated with the modified "7+3" regimen. It is noteworthy that infectious causes were responsible for all these deaths, with MDR-GNB being the predominant causative agent. This highlights the critical importance of infection management during induction therapy, particularly in patients undergoing the "7+3" regimen [24].

Comparing the outcomes of the different modified induction therapies, the "7+3" protocol demonstrated a CR rate of 55%. While the CR rate was relatively higher in this group, it was accompanied by a 20% induction mortality rate, indicating a tradeoff between efficacy and safety. On the other hand, the decitabine group had a lower CR rate of 33%, but no induction-related deaths were observed in this subgroup, suggesting a potentially safer approach for elderly or unfit patients. Our findings also revealed promising results for the venetoclax + azacytidine (Ven+Aza) group, with a 100% CR rate. Although this group consisted of only two patients, this observation warrants further investigation and potentially signifies an effective and well-tolerated induction therapy in certain AML subgroups.

Overall, our study sheds light on the challenges faced in managing AML in resource-limited settings and emphasizes the need for tailored treatment approaches based on patient characteristics and risk stratification. To improve induction therapy outcomes and reduce mortality rates, specific measures for infection prevention and management must be implemented, particularly in patients receiving the "7+3" regimen.

It is crucial to acknowledge the limitations of our study, primarily its retrospective nature, which may introduce biases and restrict data availability. Being a single-center study, the findings may be influenced by the specific characteristics and practices of our regional cancer center, limiting the generalizability of results to other healthcare settings. The small sample size, particularly in some subgroups, further adds to the potential limitations in drawing definitive conclusions from the study outcomes. Despite these limitations, our study contributes valuable insights into the outcomes of modified induction therapies in the regional cancer center setting, providing a foundation for future prospective studies with larger and more diverse cohorts. Such prospective studies could further validate our findings and potentially strengthen the clinical significance and broader applicability of the observed results.

## Conclusions

The study provides valuable insights into the outcomes of modified induction therapies for AML in a regional cancer center setting. The modified induction therapy using the 7+3 regimen showed favorable outcomes in young and fit AML patients. High-dose daunorubicin and intermediate-dose cytarabine demonstrated higher complete remission rates and improved overall survival, while HMAs emerged as viable options for elderly or unfit patients. There are limitations such as the retrospective nature of the study and the small sample size. However, our study provides scope for future prospective studies to validate these findings and explore the clinical significance in diverse patient populations.
